# The toxic effects of chloroquine and hydroxychloroquine on skeletal muscle: a systematic review and meta-analysis

**DOI:** 10.1038/s41598-021-86079-4

**Published:** 2021-03-23

**Authors:** Claudia Cristina Biguetti, Joel Ferreira Santiago Junior, Matthew William Fiedler, Mauro Toledo Marrelli, Marco Brotto

**Affiliations:** 1 Bone-Muscle Research Center, College of Nursing & Health Innovation, University of Texas-Arlington, 655 W. Mitchell Street, Arlington, TX 76010 USA; 2 Health Sciences Center. Unisagrado – Irmã Arminda, 10-50, Bauru, SP Brazil; 3 Department of Epidemiology, School of Public Health, University of São Paulo, Avenida Dr. Arnaldo 715, São Paulo, SP 01246‑904 Brazil; 4 Department of Bioengineering, University of Texas-Dallas, 800 W. Campbell Road, Richardson, TX 75080 USA

**Keywords:** Diseases, Medical research

## Abstract

The aim of this systematic review was to perform qualitative and quantitative analysis on the toxic effects of chloroquine (CQ) and hydroxychloroquine (HCQ) on skeletal muscles. We designed the study according to PRISMA guidelines. Studies for qualitative and quantitative analyses were selected according to the following inclusion criteria: English language; size of sample (> 5 patients), adult (> age of 18) patients, treated with CQ/HCQ for inflammatory diseases, and presenting and not presenting with toxic effects on skeletal muscles. We collected data published from 1990 to April 2020 using PubMed, Cochrane Library, EMBASE, and SciELO. Risk of bias for observational studies was assessed regarding the ROBIN-I scale. Studies with less than five patients (case reports) were selected for an additional qualitative analysis. We used the software Comprehensive Meta-Analysis at the confidence level of 0.05. We identified 23 studies for qualitative analysis (17 case-reports), and five studies were eligible for quantitative analysis. From case reports, 21 patients presented muscle weakness and confirmatory biopsy for CQ/HCQ induced myopathy. From observational studies, 37 patients out of 1,367 patients from five studies presented muscle weakness related to the use of CQ/HCQ, and 252 patients presented elevated levels of muscle enzymes (aldolase, creatine phosphokinase, and lactate dehydrogenase). Four studies presented data on 34 patients with confirmatory biopsy for drug-induced myopathy. No study presented randomized samples. The chronic use of CQ/HCQ may be a risk for drug-induced myopathy. There is substantiated need for proper randomized trials and controlled prospective studies needed to assess the clinical and subclinical stages of CQ/HCQ -induced muscle myopathy.

## Introduction

The use of chloroquine (CQ) and hydroxychloroquine (HCQ) have been demonstrated as effective antimalarial drugs^1^, and for use as treatments for inflammatory diseases, such as sarcoidosis^2^, rheumatoid arthritis (RA)^3^, systemic lupus erythematosus (SLE)^4^, and other inflammatory diseases^5^. CQ was approved in the United States by the Food and Drug Administration (FDA) in the 1950s for the treatment of inflammatory diseases, while HCQ, which has lower toxicity, was developed and approved 5 years later for the same purposes^6^. HCQ was subsequently preferred for long term use and, due to its lower toxicity, has been prescribed in higher doses for the treatment of these conditions^7^. However, even HCQ presents comparable side effects to CQ, such as retinopathy^7,8^, skeletal myopathy^9^, and cardiomyopathy^10^.

The pharmacologic actions of CQ/HCQ relate with their chemical interactions with cellular compartments. CQ/HCQ are weak bases and present a higher affinity to enter into acidic vesicles (lysosomes, endosomes, and autophagosomes), and the potential to neutralize these compartments^11^. CQ and HCQ are amphiphilic drugs, water and lipid-soluble, a feature that enables these drugs to pass by simple diffusion through cell and organelles membranes^12^. However, amphiphilicity only occurs at a physiologic pH (7.4). After crossing membranes of acidic organelles (lysosomes and endosomes) with low pH (4 or 5), CQ/HCQ are converted into bi-protonated molecules, becoming more acidic, making them unable to traverse back through the vesicular membrane. As a result, CQ/HCQ remains sequestered within these organelles^13^. In this way, both drugs can accumulate in the lysosomes and autophagosomes of leukocytes and consequently affect their functionality for self-antigen presentation, interfering with the production and release of pro-inflammatory cytokines in autoimmune/chronic inflammatory diseases^4^.

Off-target CQ/HCQ actions on organism may occur due to similar mechanisms related to the drug modalities for the treatment of autoimmune diseases but leading to undesirable effects depending on the cells that are affected. Due to its lysosomal affinity, CQ/HCQ can accumulate in cells from several other tissues with consequent tissue injury, such as liver^14,15^, kidney^16^, retina^8^, and skeletal^9^ and cardiac muscle cells^17,18^. Considering muscle toxicity, CQ/HCQ tissue deposits are known to induce vacuolar myopathy and the development of dense membranous structures, curvilinear and lamellar bodies, which damage muscle fibers. Curvilinear bodies are formed when CQ/HCQ deposits affect intra-lysosomal phospholipid catabolism, leading to phospholipid accumulation^16^. Another CQ/HCQ complication, autophagic vacuolar myopathy can develop via the inhibition of physiologic autophagy by lysosome disruption^19^. Physiologic autophagic processes are required for the degradation of cytoplasmic components, cellular homeostasis, and organelle turnover^20^. Lamellar and curvilinear bodies examined ultra-structurally and myocyte autophagic vacuolation observed via light microscopy are typical features from muscle biopsies required for confirmatory diagnosis of CQ/HCQ muscle induced toxicity^5,19^. However, the mechanism related to the muscle weakness is still unknown. In general, a muscle biopsy is performed on patients with clinical manifestations of myopathy combined with chronically elevated muscle enzyme disturbances, such as aldolase (ALD), creatine phosphokinase (CK or CPK) and lactate dehydrogenase (LDH)^9^.

Despite muscle toxicity being seen as a rare complication of prolonged CQ/HCQ treatment of inflammatory diseases, clinical studies have demonstrated a higher prevalence of muscle toxicity^9,10^. Tselious et al.^10^ had previously conducted a systematic review followed by descriptive statistics on antimalarial-induced cardiomyopathy and showed a mortality rate of 45% from this complication^21^. Recently, the same group reported similar clinical outcomes after CQ/HCQ discontinuation in eight geriatric women presenting CQ/HCQ-induced cardiomyopathy from their previous cohort, with a mean age 62.88 ± 9.04 presenting CQ/HCQ-induced cardiomyopathy^10^. Furthermore, a prospective longitudinal study by Casado et al.^9^ revealed a higher prevalence of CQ/HCQ-induced myopathy than previously recognized in rheumatology, with 15 out 119 patients presenting clinical and analytic antimalarial-induced skeletal myopathy, such as muscle weakness and abnormal levels of muscle enzymes (LHD and/or CK)^9^. Most of the patients (12 out 15) with CQ/HCQ-induced myopathy were women with a mean age ± SD of 63.09 ± 12.12. These studies suggest that muscle toxicity seems to be under-diagnosed in geriatric patients, possibly due to the erroneous attribution of symptoms to other age-related causes affecting cardiac and skeletal muscles, such as ischemic cardiomyopathy^10,21^ and sarcopenia^22^.

In the seventy years that CQ/HCQ have been prescribed for inflammatory diseases, the risks have been tolerated by prescribers^11^. While CQ/HCQ side effects in muscles have been considered extremely rare, recent studies suggest that these effects are likely underdiagnosed^11,21^. The same assumptions exist considering skeletal muscle toxicity^9,23^. However, no systematic reviews have investigated clinical studies about potential side effects of CQ/HCQ on skeletal muscles. To provide more evidence and useful information about the incidence and prevalence of clinical and subclinical toxic effects of CQ/HCQ in skeletal muscles, we performed a systematic review and meta-analysis based on the PICO (Population, Intervention, Comparison and Outcome) index. We considered studies with adult patients (Population), chronically treated with CQ/HCQ (Intervention), and presenting and not presenting clinical toxic effects on skeletal muscle (Comparison), in order to analyze clinical or subclinical stage (laboratory findings) of drug-induced myopathy (Outcomes).

## Results

### Systematic search in different databases and extracted key data

**Figure 1:**
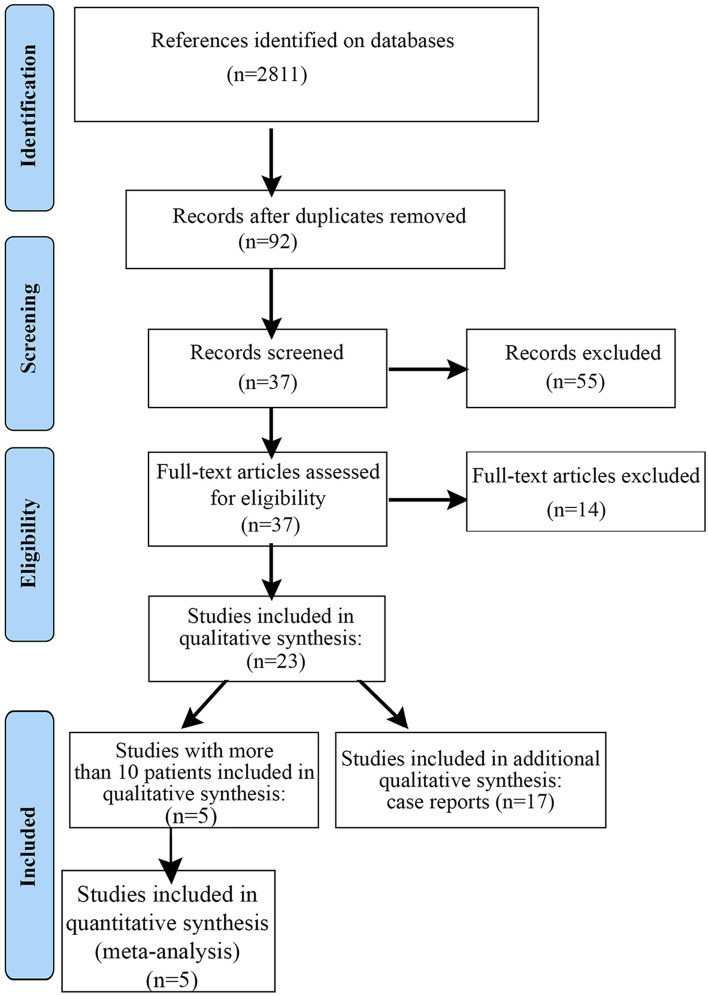
Flow diagram of the literature search strategy and eligibility.

As demonstrated in the flow diagram (Fig. 1), in our systematic and exploratory search we identified 2,811 references, filtered according to articles titles and abstracts to 92 references. After removing duplicates and applying inclusion and exclusion criteria, 37 full-text articles were assessed for eligibility, resulting in 14 observational studies with more than 5 patients, 5 studies eligible for qualitative and quantitative analysis. Full-texts were excluded due to the following reasons: evaluation of skeletal muscle function considering the effects of inflammatory underlying diseases and not the treatment with CQ/HCQ^24–26^, less than 10 patients treated with CQ/HCQ^27–30^; symptoms of muscle weakness or myalgia are potentially related to the primary disease (polymyositis and dermatomyositis)^26,31^. The study of Lee et al.^19^ was excluded from the qualitative and quantitative analysis, since the aim of this study was to investigate markers to be expressed in autophagic vacuolar myopathy induced by HCQ. Studies exclusively related to drug-induced cardiomyopathy were excluded from this analysis. No inter-reviewer disagreements were found between reviewers (CB and MM). Other co-authors reviewed the selected articles to confirm their inclusion (MF and JFSJR). Author MB completed a final review.

### Qualitative analysis of eligible observational studies

The five studies included in the systematic review were published from 1999 to 2017. Tables 1 and 2 summarize the major information collected from these studies in the systematic review. The selected studies were classified as prospective^9,32,33^ and retrospective case controls^5,23^. The levels of evidence found in these studies were III-2 and III-3. Two studies were from Canada^23,32^, one from Spain^9^, one from the United States^33^, and one from Australia^5^ (Table 1). The range of period for continuous CQ/HCQ treatment was a minimum of 3 months and a maximum of 9 years for 5 studies. The range of mean age identified in these studies were: 34 ± 14 years for Wang et al.^32^; and 41.85 ± 14.45 (group case, a), 43.86 ± 15.95 (group control, b) for Tselios et al.^23^; 51.67 ± 11.37 years for Kalajian et al.^33^ (2009); 57 ± 13.9 years for Casado et al.^9^ Khoo et al.^5^ did not report the mean age of patients, and regarding patient sex^9,23,32,33^, the majority were women. The reported primary diseases requiring the long term treatment with CQ/HCQ were SLE^9,23,32^ and other variants of lupus^9,33^, RA and other related rheumatic conditions^9^ (Table 1). The study of Wang et al.^32^ presents several side effects or other reasons (e.g., lack of efficacy, pregnancy) for discontinuation of antimalarials by course of treatment in 156 patients with SLE. This study presented a detailed data survey from all patients analyzed, including average dose (mg/Kg/day) associated with each side effect. Wang et al.^32^ reported 3 pregnant patients in the follow up for antimarial discontinuation among the 224 patients, but drug discontinuation was not related with muscle myopathy.

**Table 1:** Data summary for the six studies selected for CQ/HCQ-induced myopathy.

Authors	Year	Country	Type of study/Level of evidence*	Total #Patients (genders F, M)	Patients treated with HCQ/CQ	Patient age in years mean/SD	Dosages (mg for daily dose; gr for cumulative dose)	Duration (years mean or mean range)	Primary diseases
Wang et al.^32^	1999	Canada	Prospective study/Level III-2	224 (136F, 88NR)	156 HCQ or CQ	34 ± 14	200 to 400 mg/day HCQ	4.2 ± 5.6	SLE (number of patients)
Casado et al.^9^	2005	Spain	Prospective study/Level III-2	119 (84 F, 35 M)	111 CQ/8 HCQ	57.5 ± 13.9	3.5 mg/kg/day QC, 6.5 mg/kg/day HQC	3 (40.4 months)	RA [69], PR [14], SS [11], SLE [9], , CT [7], psoriatic arthritis [4], and other rheumatic conditions [5]
Kalajian et al.^33^	2009	USA	Prospective study/Level III-2	21 (20 F, 1 M)	8 CQ/13 HCQ	51. 7 ± 11.37	minimum of 200 mg of HQC or 250 mg of QC/daily	0.25–9.0	LE
Tselios et al.^23^*	2016	Canada	Retrospective Case Control study/Level III-3	1778 87.1% F (cases), 87.8% F (controls)	186CQ/806 HCQ**#**	41.85 ± 14.45a 43.86 ± 15.95b	155.11gr of CQ (a) 341.55gr of HCQ (a) 89gr of CQ (b) 355gr of HCQ (b)	3.84–4.33	SLE
Khoo et al.^5^	2017	Australia	Retrospective Case Control study/Level III-3	34 (NR)	18	NR	200 mg–800 mg	0.35–11.58	Inflammatory Myositis

*F* female, *M* male; Primary diseases: LE (various types of lupus Erythematosus), *SLE* systemic lupus erythematosus, *RA* rheumatoid arthritis, *PR* palindromic rheumatism, *SS* Sjogren’s syndrome, *CT* connective tissue disease.

^#^Sum of group case + group control (group case are patients with elevated enzymes for muscle injury (CK) and group control is patients with non-elevation. a = case group b = control group.

*Data from Tselios et al.^23^ were obtained from Table 1 of the original study.

**Table 2:** Qualitative data for the six studies selected for CQ/HCQ-induced myopathy.

Authors	Year	Patients treated with HCQ/CQ	Number of patients with clinical symptoms of muscle toxicity	Number of patients with biopsy for drug induced myopathy	Number of patients with elevated muscle enzymes (CK or LHD)	Intervention after diagnosis of muscle toxicity	Outcome after drug discontinuation
Wang et al.^32^	1999	156 HCQ or CQ	2	1	0	Drug discontinuation	Both patients clinically improved after discontinuation (2 months)
Casado et al.^9^	2005	111 CQ/8HCQ	15	15	22	Drug discontinuation in 7 patients	Signs and symptoms disappeared for 5 of 7 patient, one NR and 1 diminished
Kalajian et al.^33^	2009	8 CQ/13 HCQ	7	NR	4	Suggest discontinuation, but NR	NR
Tselios et al.^23^	2016	184CQ/853 HCQ	5	0	216	203 patients were followed for 7.3 ± 5.6 years: 49.8% had persistent and 14.8% intermittent CPK elevation	No drug discontinuation reported
Khoo et al.^5^	2017	34 HCQ	18 (10*)	18	10	N/A	N/A

In Casado et al.^9^, 22 patients were reported with elevated muscle enzymes, but only 15 patients are present with complete follow-up information for clinical symptoms. Regarding the study of Tselios et al.^23^, the number of patients treated with HCQ or CQ were extracted from a total of 1,037 patients. Symbol (*) indicate patients presenting muscle weakness and without histological features/markers positive for active inflammatory myositis (primary disease).

Concerning the patients with symptoms of muscle weakness and related with CQ/HCQ muscle toxicity, 37 patients of 1,367 from five studies presented relevant clinical manifestations of proximal muscle weakness^5,9,23,32,33^. Drugs were discontinued in 7 from 22 patients with severe muscle weakness in Casado et al.^9^, with complete recovery for 5 patients. In Wang et al.^32^, the 2 patients presenting severe proximal muscle weakness also presented a significant clinical improvement 2 months after antimalarial discontinuation. During the study, these authors reported 21 deaths, none related to the use of CQ/HCQ. Considering the biopsy of skeletal muscles for CQ/HCQ induced-myopathy, 3 studies presented data of histopathological findings^5,9,32^, including typical autophagic vacuolar changes^9,32^, curvilinear and lamellar bodies in ultrastructural examination^5,9^. Khoo et al.^5^ observed no correlation with the detection of curvilinear bodies with cumulative dosing of HCQ in terms of body weight or body mass index. Also, in the study of Khoo et al.^5^, the authors described 67% of patients with curvilinear bodies received co-prescription of proton-pump inhibitors (PPI). The elevation of muscle enzymes was reported in four studies^5,9,23,33^.

## Quantitative analysis

### Outcome results are described following secondary/individual outcomes

#### Reported clinical symptoms of CQ/HCQ related muscle toxicity

**Figure 2:**
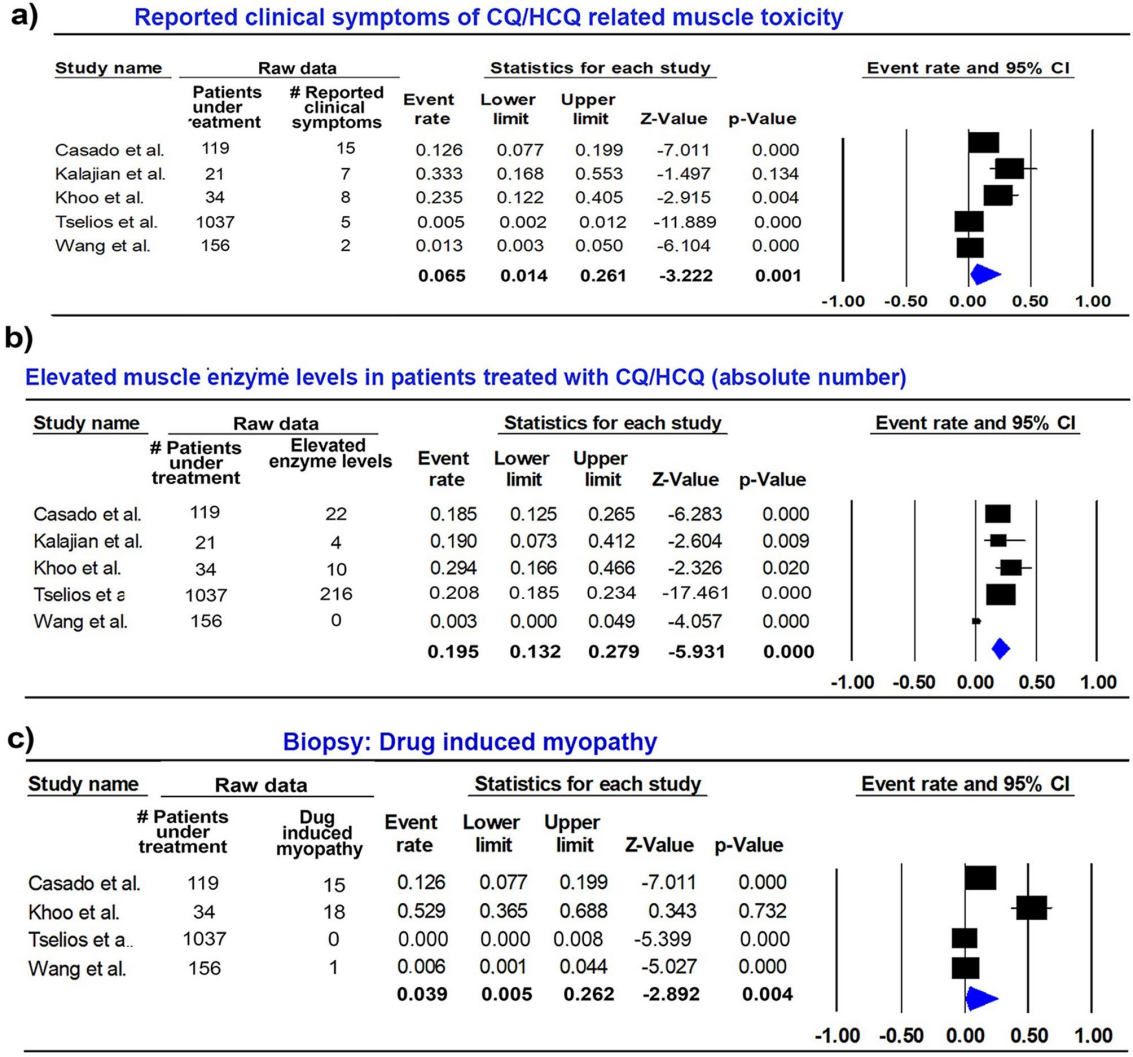
(**a**) Forest plot for variable: reported clinic symptoms of QC/HQC related muscle toxicity. (**b**) Forest plot for variable: Laboratorial findings of elevated muscle enzyme levels in patients treated with QC/HQC. (**c**) Forest plot for variable: Confirmatory Biopsy for Drug-induced myopathy. *CI* confidence interval.

Five studies involving 1,367 patients treated with QC/HCQ were analyzed for symptoms of muscle toxicity. Data from event ranged from 1.4% to 26.1%. The overall pooled for event rate was 6.5% (random; 95% CI 1.4% to 26.1%; Fig. 2a). Reported clinical symptom data presented a high heterogeneity (p = 0.000, I^2^ = 94.695).

#### Laboratory findings of elevated muscle enzyme levels in patients treated with CQ/HCQ

In this analysis, three studies involving a total of 1,367 patients treated with CQ/HCQ were included. Data from event ranged from 13.2% to 27.9%. The overall pooled for event rate was 19.5% (random; 95% CI: 13.2% to 27.9%; Fig. 2b). The heterogeneity for laboratory findings was considered moderate (p = 0.021, I^2^ = 65.533).

### Biopsy for CQ/HCQ-induced myopathy

In this analysis, considering 1,346 patients treated with QC/HQC, data from event ranged from 0.5% to 26.2%. The overall pooled for event rate was 3.9% (random; 95% CI: 0.5% to 26.2%; Fig. 2c). The heterogeneity for the data outcome biopsy for drug-induced myopathy was considered elevated (p = 0.000, I^2^ = 94.632).

### Study quality and risk of bias for observational studies

Of the five observational studies evaluated for qualitative and for meta-analysis, only three used a prospective follow-up composition^9,32,33^. It is worth mentioning that a study had a greater laboratory focus not allowing a follow-up of important clinical variables^5^. The ROBINS-I scale allowed an evaluation in the 7 domains indicating; possible biases in the pre-intervention, relating to the composition of the samples, withdrawal and exclusion of patients, absence of sample calculation, and confounding variables that can influence the results (Domain 1: Bias due to confounding; Domain 2: Bias in selection of participants); the studies adequately delimited the interventions without presenting deviations in interventions based on the use of medications in longitudinal studies (Domain 3: Bias in classification of intervention/Domain 4: Bias due to deviations intended from interventions).

**Figure 3:**
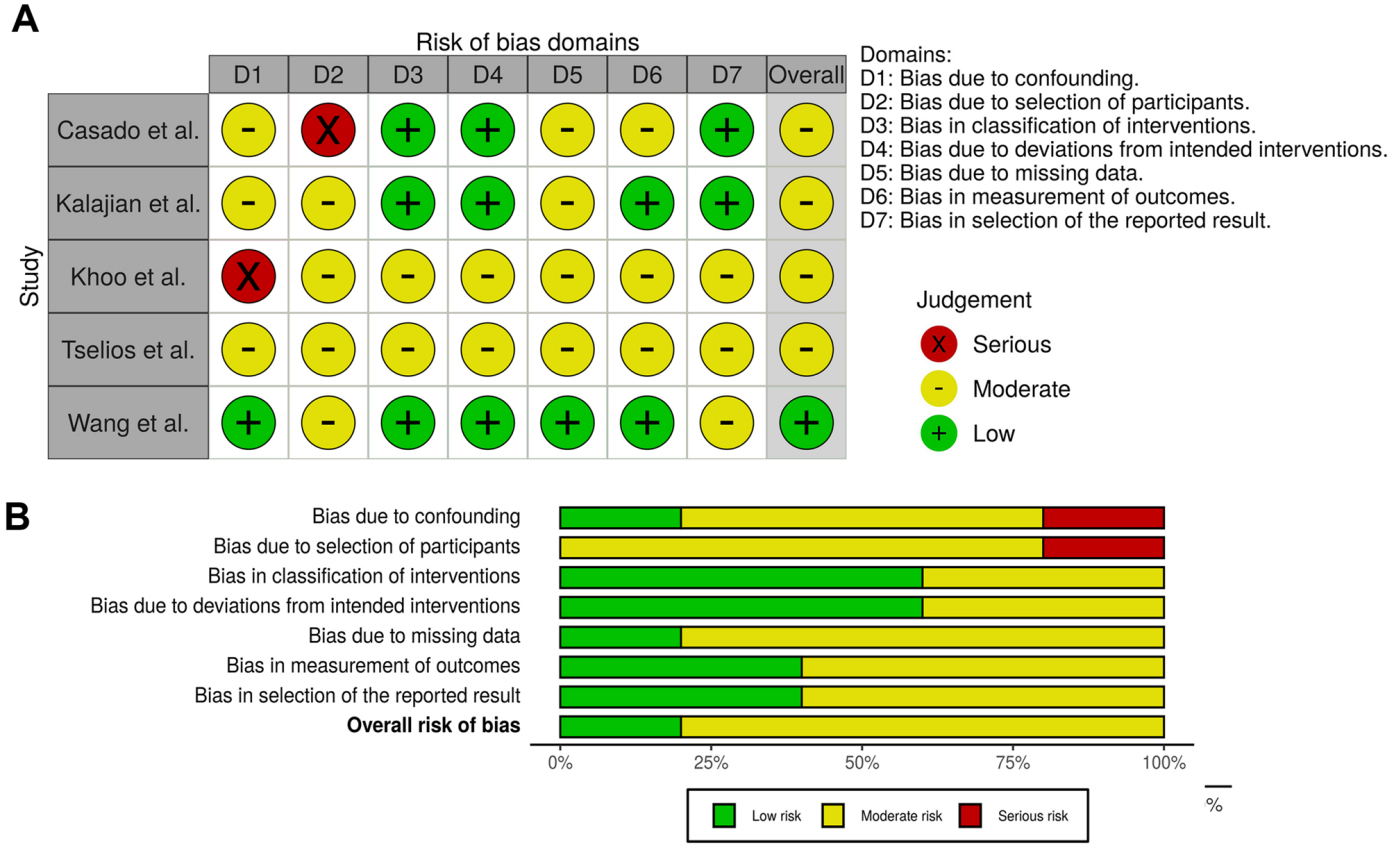
(**A**) Bias domains in ROBINS-I for included studies. (**B**) Total Score for each domain (ROBINS-I).

There were indications of data loss in relation to the sample of patients or deficient collection of information in some studies (Domain 5: Bias due to missing data). In relation to the measurement of the results, there was similarity in the analyzed groups (Domain 6: Bias in measurement of outcomes). However, there was exclusion of patients in specific analyses. Regarding the dissemination of results (Domain 7: Bias in selection of the reported results), the studies did not present the main results in a similar way and this item may have been impaired in non-prospective studies. In general, we opted for a cautious and conservative analysis in studies focused only on pathological and retrospective data (cohort and case controls) as it is not possible to evaluate the construction of samples, the establishment of the evaluated outcomes, the disclosure of timely results, some drugs administered systemically, as well as complete information of the systemic state of the patient may have been undervalued. Finally, it is worth noting that no study randomized the sample. Individual and general data of the studies can be observed in Fig. 3A,B.

### Additional qualitative table with case reports

In order to carry out an analysis of the case reports, the relevant studies in the area that were in accordance with the PICO criterion and inclusion criteria, but with less than five patients, were included in a table for case-reports. Twenty-three full texts had less than five patients and/or were individual case reports and were consequently excluded from meta-analysis. From these 23 studies, 17 full texts involving 21 adult patients with CQ/HCQ induced myopathy were summarized in an additional descriptive table of case reports (Supplementary Table S1), due the relevance of this topic and absence of more clinical studies addressing these side effects. As demonstrated in Table S1, most of patients presenting CQ/HCQ-induced myopathy were women (17 of 21 patients), mean age of 59.88 ± 14.19 for women and 51.00 ± 14.17 for men. All 21 patients presented moderate to severe muscle weakness, 18 patients with confirmatory muscle biopsies for CQ/HCQ induced toxicity and only 2 patients had no significant abnormalities. The elevated levels of muscle enzyme (mainly CK reported) was elevated in 10 patients, normal in 4 case-reports, and not reported (NR) in 6 studies. Most patients presented a satisfactory muscle function recovery after drug discontinuation and four patients died as observed in the case reports.

## Discussion

Despite the use of CQ/HCQ worldwide for the last 70 years to treat several inflammatory conditions, few studies have focused on CQ/HCQ induced myopathy. A recent systematic review has demonstrated the prevalence of CQ/HCQ induced cardiomyopathy in elderly women^21^ and a complete follow-up of 8 patients from this study^10^. However, no systematic review and/or meta-analysis considering CQ/HCQ-induced skeletal myopathy have been published to date. In this systematic review and meta-analysis, we focused on toxic effects affecting skeletal muscles possibly associated with the use of CQ/HCQ in patients who have received systemic treatment of the drugs.

In our systematic search, we found 5 different clinical studies addressing CQ/HCQ–induced myopathy in skeletal muscles (Tables 1 and 2) and 17 case reports (Table S1). The 5 different observational studies presented a minimum of 21^12^ and a maximum of 1307^23^ adult patients under treatment with CQ/HCQ for chronic inflammatory diseases^9,23,32,33^. Importantly, one of these studies were more focused in laboratory findings and biopsies than in allowing a follow-up of important clinical variables^4^. In accordance with NHMRC classifications, the levels of evidence found in these six studies were lower (III-2 and III-3). The ROBINS-I scale was used for risk of bias (low, moderate, and serious) for evaluation in the 7 domains, as described in the results (Fig. 3). The selected studies effectively delimited the interventions without presenting deviations in interventions based on the use of medications in longitudinal studies. However, as already reported, there are limitations present in the studies, which are inherent to the experimental design; the difficulty of organizing a standardized sample is understood (reducing selection bias, confusion bias). It highlights the importance that large prospective clinical studies or randomized and controlled studies with adequate sample calculation and specific interventions can be designed in the coming years. Therefore, the quantitative data must be carefully extrapolated, but they show the current clinical reality for the proposed situation.

In our meta-analysis we evaluated three outcomes: (1) proximal or generalized muscle weakness as reported clinical symptoms of CQ/HCQ related muscle toxicity (Fig. 2a), (2) laboratory findings considering raised muscle enzyme levels (CK, LDH and/or ADL) in patients treated with CQ/HCQ (Fig. 2b), and (3) biopsy with evidence of CQ/HCQ-induced myopathy (Fig. 2c). The heterogeneity in the studies for muscle weakness and biopsy were high, while the heterogeneity for laboratory findings (CK levels) was moderate, this is due to the variability of the event rates for each study, the sample size, direction of effect, confidence interval of the results, as well as the significant weight of each study for the result of the analysis. It is therefore justified that new studies are delineated using sample calculation, randomization and using blinding concepts in order to study the subject. Previous studies have discussed these parameters to determine the diagnosis of muscle toxicity caused by CQ/HCQ in clinical and subclinical stages, and are still controversial^5,9,23^. A positive biopsy for curvilinear and/or lamellar bodies is not necessarily associated with muscle symptoms^9^ or with cumulative dosages of antimalarial drugs^5^. However, it might indicate a subclinical stage of muscle toxicity. Indeed, Casado et al.^9^ considered antimalarial myopathy in patients presenting positive microscopical findings (curvilinear bodies and/or vacuolar myopathy) associated with persistent muscle enzyme disturbance, regardless of patient clinical symptoms^9^. Importantly, in the study of Khoo et al.^5^, 67% of patients with curvilinear bodies received co-prescription of proton-pump inhibitors (PPI), suggesting that co-prescription of HCQ and PPI agents may disrupt lysosomal function and adversely affect muscle function.

Considering muscle weakness or the clinical stage of drug-induced myopathy, our qualitative analysis revealed that 37 patients out of 1,367 from 5 studies presented relevant muscle symptoms related with CQ/HCQ muscle toxicity^5,9,23^ (Tables 1 and 2). Casado et al.^9^ reported 6.7% with clinical CQ/HCQ-induced myopathy, from an accumulated prevalence of 12.6% CQ/HCQ-induced myopathy^9^. On the other hand, Wang et al.^32^ reported only 2 cases of HCQ-induced myopathy in 156 SLE patients. However, in this prospective study, authors evaluated many reasons for the 156 patients with CQ/HCQ discontinuation (e.g. remission of lupus symptoms, ocular and auditory toxicity, headache, lack of efficacy, gastrointestinal tract effects) and not necessarily the development of other stages of muscle myopathy, such as moderate muscle weakness and subclinical stages. The 2 patients that reported CQ/HCQ-myopathy developed severe muscle weakness, requiring hospitalization.

**Figure 4:**
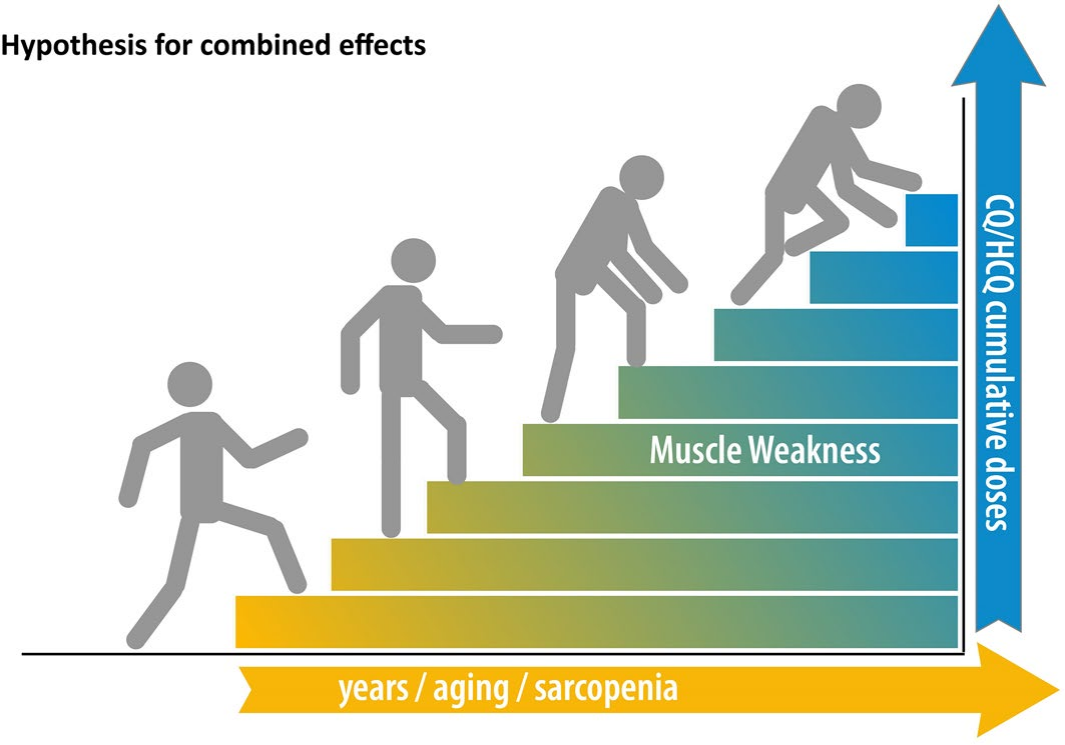
Hypothesis for combined effects of sarcopenia and CQ/HCQ induced myopathy along with aging and chronic use of drugs.

Interestingly, most of the patients from the clinical studies are over 50 years old and women, and in agreement, the case reports of CQ/HCQ-induced myopathy demonstrated 17 from 21 case reports in women, and the mean age of 59.88 ± 14.19 for women and 51.00 ± 14.17 for men. Generalized and proximal muscle weakness have been suggested asunder-diagnosed symptoms of CQ/HCQ-muscle myopathy in elderly patients, perhaps due to other underlying diseases causing muscle weakness and age, such as sarcopenia^22^. Importantly, the average age of patients affected by drug-induced myopathy is coincident with the population developing sarcopenia, a common and under-diagnosed condition that leads to loss of muscle quality during aging, and is also characterized by a decline in muscle force and power^34,35^. As a hypothesis to be investigated (regression analysis correlating age with patient symptoms), sarcopenia may be more than a confounding factor of drug-induced muscle weakness in these patients, but a co-factor affecting the overall quality of the muscle and causing a decrease in muscle strength/power in these populations (Fig. 4). Furthermore, this combination of factors could affect overall functional capacity and CQ/HCQ post-discontinuation recovery.

Of note, a drug-induced myopathy or myotoxicity is defined as the manifestation of myopathic signals and symptoms, such as CK elevation and muscle weakness, in patients without muscle disease when exposed to certain drugs^36^. However, in certain articles of this systematic review, muscle diseases were a confounding co-factor of muscle weakness related to CQ/HCQ-induced myopathy, such as the primary inflammatory disease, especially the inflammatory myopathies (polymyositis, dermatomyositis, inclusion body myositis, not otherwise specified and necrotizing myopathy)^5^ and requires the prescription of CQ/HCQ. In Khoo et al.^5^, authors evaluated muscle biopsies with curvilinear bodies from CQ/HCQ-induced myopathy in patients with different types of inflammatory myositis as the primary disease^5^. To provide a differential diagnosis of muscle weakness related with inflammatory myositis or CQ/HCQ-induced myopathy, the authors evaluated muscle inflammation biomarkers related to the primary disease (MHC I and II expression), and 10 out of 18 patients presenting muscle weakness did not present signals of active inflammatory myositis. Importantly, curvilinear bodies were found as a typical indicator of CQ/HCQ-induced myopathy in the biopsies of 18 patients, but unrelated to the cumulative dose of HCQ, while 16 patients with inflammatory myositis and treated with HCQ did not present drug-induced myopathies^5^. In this previous study, the presence of curvilinear bodies was significantly associated with deleterious effects on muscle function (proximal weakness in the absence of active inflammatory myositis). Casado et al.^9^ also demonstrated the presence of vacuolar myopathy with atrophy of type 1 and type 2 muscle fibers, and curvilinear bodies seen in a significant proportion of muscle biopsies from CQ/HCQ treated patients^9^. In addition, in another study not included in this systematic review, Lee et al.^19^, provided two additional markers, LC3 (a marker of autophagosome formation) and p62 (adapter protein involved in lysosomal degradation), to be expressed in autophagic vacuolar myopathy induced by HCQ.

Regarding the outcomes of laboratory findings (levels of CK, ALD and/or LDH), our meta-analysis demonstrated a low heterogeneity when comparing the selected articles and pooled samples, and the articles suggested that this is a critical finding to be evaluated in patients under diagnosis of drug-induced myopathy. CK elevation was positive in 10 case reports, normal 4 case-reports, and not reported (NR) in 5 studies (Table 2). Considering the observational studies, Tselious et al.^23^ reported the use of CQ/HCQ as a risk factor for elevated levels of CK in patients being treated for SLE, with a total frequency of 16.3%. Only 5 patients (2.46%) developed muscle weakness at the time of study, from a total of 203 patients presenting persistent CK elevation (considering patients who complete a follow up of 7.3 ± 5.6 years). Only one of these 5 patients had a muscle biopsy, with no positive findings of drug-induced myopathy, and one patient developed HCQ-induced cardiomyopathy with confirmatory biopsy^23^. In Khoo et al.^5^, 64% of the patients under treatment for inflammatory myopathies, and positive biopsies for HCQ-induced myopathy had elevated CK levels, reinforcing that persistence in muscle enzyme disturbance is a complementary can represent a complementary finding of drug-induced myopathy^5^. In agreement, Casado et al.^9^ demonstrated a persistent muscle enzyme disturbance in 18.5% (22 of 119 patients) under subclinical or clinical stages of CQ/HCQ-induced myopathy^9^. Specifically, the authors demonstrated elevated levels of LDH for 19 of 22 patients, CK for 7 of 22 patients and ADL for 3 of 22 patients^9^. Kalajian et al.^33^ showed a higher prevalence of muscle enzyme elevation (CK, LDH and/or ADL) in CQ-treated patients (38%) than HCQ-treated patients (8%).

Importantly, CK is more specific for muscle injury/muscle diseases compared to ADL and LDH, thus elevation of this particular enzyme is part of the criteria for diagnosis in patients exposed to myotoxic drugs, but it is a complementary approach and not sufficient for confirming the diagnosis of toxic myopathy^36^. It is important to emphasize that LDH and ADL are present in other tissues (liver and brain), and are useful markers to analyze liver function, being elevated in patients in serum of patients with hepatic injury^37^. Also, elevated levels of LDH might be considered in patients with inflammatory connective diseases, such as RA and under treatment with anti-inflammatory drugs^38^. As aforementioned, CQ/HCQ can also accumulate in liver and cardiac cells, causing hepatotoxicity^15^ and cardiomyotoxicity^10,21^. Thus, it is reasonable to affirm that the serum elevation of these enzymes in patients under prolonged treatment with CQ/HCQ may also indicate liver and cardiac muscle damage.

Considering the outcomes of antimalarial myopathy, from the elected studies, Wang et al.^32^ and Casado et al.^9^ reported therapy discontinuation for 2 and 7 patients with drug-induced myopathy, respectively. Most of the patients had a complete recovery or significant remission of muscle weakness. As a limitation, the other 4 selected studies did not report the patient’s follow-up or drug discontinuation. We also added 17 case reports, considering the scarcity of information from literature and the relevance of outcomes described for patients with CQ/HCQ-induced myopathy (Table S1). The improvement period for muscle symptoms varied from 2 weeks^39^ to 9 months^42^. Normalization of muscle enzyme levels was also detected in some case reports after CQ/HCQ discontinuation^40–42^. Importantly, the half-life of CQ/HCQ is about 40–50 days^43^ and symptom remission may not begin until a significant amount of CQ/HCQ has been eliminated from the body.

This systematic review sought to follow international standards for this type of study, including submission to PROSPERO, monitoring of the PRISMA statement, Protocol and Abstract guides. All searches and analyzes were double checked and underwent a final review and systematically passed each statistical step required of both a Systematic and a Meta-Analysis. Upcoming updates should expand the quantitative qualitative analysis and seeking standardized clinical studies.

Finally, concerns about the potential toxic effects of CQ/HCQ have gained more attention after the inclusion of these drugs in clinical trials for the prevention and treatment of SARS-CoV-2 infection (COVID-19) several groups around the world are engaged in these important analyses, and careful analysis may be necessary especially considering elderly patients^44^. We believe the information provided by this review should be considered only when CQ/HCQ therapy is indicated as a chronic treatment for inflammatory diseases (PICO issue of this systematic review). However, caution is to be expected in the recommendations to the recent indications for testing COVID-19 patients in clinical trials, such as for high-risk groups including aged and sarcopenic patients.

## Conclusion

In conclusion, our findings draw attention to CQ/HCQ muscle toxicity as an under-diagnosed side effect, promoting the need for new clinical studies considering the population under chronic treatment with CQ/HCQ^11^. As demonstrated by our findings, the idea of lower incidence of CQ/HCQ (less than 2%) is from few studies or case reports with low levels of evidence^32,33^. More recent studies, however, have indicated a higher incidence and prevalence of clinical or subclinical stages of CQ/HCQ-induced myopathy^9,23^. It is essential that clinical studies with strict protocols are designed in the coming years. This systematic review and meta-analysis study confirms the complexity of reaching universal conclusions based only on simpler literature reviews even for a topic represented by almost 3,000 papers. It is important to draw more solid conclusions, particularly for critical clinical decisions from solid, unbiased systematic reviews.

## Material and methods

### Standard criteria and type of study

This systematic review and meta-analysis were designed according to the criteria established by the Cochrane Collaboration for the elaboration of systematic review and meta-analysis. Thus, this study was followed by PRISMA-P and PRISMA Statement to ensure the standardization of the data inclusion/exclusion criteria and analysis, as previously described in other studies^45,46^.

### Protocol and registration by PROSPERO database

This systematic review and meta-analysis were registered in the PROSPERO database under number CRD42020183677. This data will be available with the project title “The toxic effects of chloroquine and hydroxychloroquine on skeletal muscle—a systematic review and meta-analysis”. The authors followed the PRISMA-P protocol for planning a systematic review^45,46^.

### Eligibility criteria

The researchers conducted analyzes based on the PICO index:

- *Population* Patients in the published reports that were submitted to the treatment of chronic diseases.
- *Intervention* The use of CQ or HCQ.
- *Comparison* Patients treated with CQ or HCQ presenting and not presenting adverse effects on skeletal muscle.
- *Outcome* Results of assessing the muscular complications of both drugs, considering symptoms, laboratory findings and/or confirmatory biopsies of drug-induced myopathy.

### Inclusion criteria for meta-analysis

The studies were selected according to the search strategy respecting the inclusion criteria. The inclusion criteria were: (1) English language; (2) Clinical follow-up studies of at least 6 months of the types: retrospective, prospective, and controlled and randomized clinical trials; (3) Period analyzed from 1990 to 2020; (4) Adults > age of 18; (5) studies with more than 10 patients.

### Exclusion criteria

Exclusion criteria for meta-analysis was applied to pre-clinical in vitro and animal studies, clinical studies presenting uncontrolled clinical cases, with less than 5 patients or with incomplete data, pregnancy, children, and adolescents, and studies only related to cardiomyopathies. Other reviews and studies that did not allow the collection of the necessary information were disregarded and excluded.

### Additional qualitative table with case reports data

Excluded case reports (articles with less than 10 patients) were not considered as high evidence for meta-analysis but were included for the qualitative analysis. We used this information including studies in English language, from 1990 to 2020, with adult patients (> 18 years old), who received CQ/HCQ and developed serious musculoskeletal side effects.

### Study search strategy

The databases used were Medline/PubMed, Cochrane Library, EMBASE, and SciELO. Searches were performed for articles published until April 1, 2020. Additional contact was made with the authors when it was not possible to find the article via the national online system or COMUT.

### Searches: keywords and data bases

The keywords based on MeSH/PubMed were: “chloroquine diphosphate”, “Muscular Diseases”, “Myotoxicity”, “chloroquine”, “Skeletal Muscle”, “myopathy”, “Hydroxychloroquine”. The MeSH terms and/or text words were combined by “AND” or “OR” in each domain. A manual search was also carried out in the specific journals in the area: The Journal of Rheumatology, and Lupus.

### Data collection process

This research was developed by previously calibrated researchers, the selection of articles and data collection was performed by a previously calibrated reviewer (CB) and another reviewer (MM). Both were advised by a third reviewer (JFSJR) in the event of a discrepancy. MB conducted a final review. Meetings to consensus the selection of each article in the sample were scheduled in order to eliminate disagreements.

### Items to be extracted

The data extracted from each study were analyzed and main information obtained in an orderly and standardized manner. The following data were collected for the articles: authors, year, type of study, total of patients, country, average age, patients treated with CQ/HCQ, control group, dosages, duration, primary disease, reported muscle symptoms of toxicity, biopsy, drug induced myopathy, laboratory findings (CPK or CK levels), intervention findings, intervention after diagnosis (drug discontinuation or not), possible outcomes after drug discontinuation, number of deaths, and main conclusion of articles. All data collected by one reviewer (CB) was verified by another reviewer (MM). Data collection was performed using an Excel spreadsheet (Microsoft, Washington, USA).

### Assessment of study quality and risk of bias

The included clinical studies were evaluated in relation to the classification of the type of clinical study, being retrospective, clinical case series, prospective or randomized controlled trials, as indicated by The National Health and Medical Research Council (NHMRC. 2000). In addition, it was applied the ROBINS-I (Risk Of Bias In Non-randomized Studies of Interventions) scale to the Non-Randomized studies of the effects of interventions^47^. This scale was developed by members of the Cochrane Bias Methods Group and the Cochrane Non-Randomized Studies Methods Group (available from http://www.riskofbias.info). The group used the tool Robvis, available online: https://mcguinlu.shinyapps.io/robvis/, in order to elaborate answers for the seven domains^48^.

### Summary of measures used and statistical analysis

Data were grouped considering the following variables/outcomes: (1) Reported clinical symptoms of CQ/HCQ related to muscle toxicity; (2) laboratory findings of elevated muscle enzyme levels (ALD, CK and/or LDH) in patients treated with CQ/HCQ and presenting or not with drug-induced myopathy; and (3) confirmatory biopsy for drug-induced myopathy in patients treated with CQ/HCQ. These data were tabulated allowing analysis of risk ratio (RR) with 95% CI (confidence interval), the contribution weight of each study was performed to calculate the meta-analysis. The dichotomous data were analyzed using RR and 95% CI. For all analyzes performed, significant values were *p* < 0.05. The software and Comprehensive Meta-Analysis Software (Software version 3.0 -Biostat, Englewood, NJ, USA) was used for meta-analysis and forest plot graphs^49^(Figs. 1–3). Figure 4 was originally drawn in Adobe Creative Cloud 2020.

Total number of patients treated with CQ/HCQ was considered as statistical unit. The complication rate (CR) of CQ/HCQ treatment was pooled and were reported as event rate using the Mantel–Haenszel test with 95% CI.

### Outcomes

#### Primary outcome

Analyze the side effects on skeletal muscles possibly associated with the use of CQ/HCQ in patients who have received systemic chronic treatment of the drug.

#### Secondary outcomes

1. Reported clinical symptoms of CQ/HCQ related to muscle toxicity.
2. Laboratory findings of elevated muscle enzyme levels (ALD, CK and LDH) in patients treated with CQ/HCQ and presenting or not with drug-induced myopathy.
3. Confirmatory biopsy for drug-induced myopathy in patients treated with CQ/HCQ.

### Bias risks in quantitative data

Heterogeneity was assessed using the Q (× 2) method and the value of I^2^ was measured. The statistical value of I^2^was used to analyze the percentage of total variation across studies due to heterogeneity^50,51^. I^2^ values higher than 75 (range 0–100) may indicate a relevant heterogeneity as demonstrated in previous studies^50,52–54^, thus we adopted a random effect in these conditions, since the studies demonstrated different characteristics when compared, such as sample size, data from different type of groups, localization. Peculiarities of each experimental design from each study were also evaluated.

### Additional analysis

The analyses of secondary outcomes was considered in order to complement the initial analysis, sensitivity tests for subgroup analysis were performed to avoid any potential for heterogeneity^52,54^.

### Ethical approval

Data inserted in this article were obtained from existing publications, thus Ethical approval was not applicable for this study.

## Supplementary information

Supplementary information.

## Data Availability

Data analyzed during this study are included in the published selected articles and its additional files.
