# Supplementary material for: The toxic effects of chloroquine and hydroxychloroquine on skeletal muscle: a systematic review and meta-analysis
Source: Sci Rep. 2021 Mar 23;11:6589. doi: 10.1038/s41598-021-86079-4 (PMC7988151; doi:10.1038/s41598-021-86079-4)
Supplement: Supplementary file 1 — Supplementary information. [file 41598_2021_86079_MOESM1_ESM.docx]

**The toxic effects of chloroquine and hydroxychloroquine on skeletal muscle – a systematic review and meta-analysis**

Claudia Cristina Biguetti^1^, Joel Ferreira Santiago Junior^2^, Matthew William Fiedler^1^, Mauro Toledo Marrelli^1,3^ and Marco Brotto^1^

^1^Bone-Muscle Research Center, College of Nursing & Health Innovation, University of Texas-Arlington, 655 W. Mitchell Street, Arlington, TX 76010, USA

^2^Health Sciences Center. Unisagrado – Irmã Arminda, 10-50, - Bauru - SP - Brazil

^3^Department of Epidemiology, School of Public Health, University of São

Paulo, Avenida Dr. Arnaldo 715, São Paulo, SP 01246‑904, Brazil

**Supplementary Information**

**Table S1.** Case reports of CQ/HCQ-induced myopathy (1990-2020)

| **Authors** | **Year** | **Age gender** | **Drug and Dose (mg/daily)** | **Duration (years)** | **Underlying**  **Disease** | **Muscle biopsy** | **CK levels** | **Outcome** |
| --- | --- | --- | --- | --- | --- | --- | --- | --- |
| Avina-Zubieta *et al.**^55^ | 1995 | 40, M | CQ, 250 | 1.4 | RA | No significant abnormalities | Normal | Symptoms recovery after 2m of QC discontinuation |
| Avina-Zubieta *et al*.*^55^ | 1995 | 71, M | CQ, 250 | 1.4 | RA | Type II fiber atrophy  Rare rimmed vacuoles |  |  |
| Avina-Zubieta *et al*.*^55^ | 1995 | 49, F | CQ, 250 | 1.4 | RA | Rare necrotic fibers  Moderate fiber variability |  |  |
| Nucci *et al*.^56^ | 1996 | 59, F | CQ, 250 | 0.16 | RA | Type II fiber atrophy and rimmed vacuoles | Normal | No significant regression of symptoms/ patient died |
| Richards^40^ | 1998 | 65, F | HCQ, 400 | 6 | SLE | No significant abnormalities | Elevated | CK normal levels after 17 days of QC discontinuation |
| Richter *et al*.^41^ | 2003 | 57,F | CQ, NR | NR | SLE | Vacuolar degeneration  curvilinear bodies profile | Elevated | CQ was discontinued CK levels returned to normal |
| Finsterer, Jarius[^57^](#_ENREF_48) | 2003 | 81, F | CQ, 250 | 6 | CPA | fiber atrophy  vacuolar myopathy | NR | Symptoms recovery within 5 m after CQ discontinuation |
| Bolaños-Meade *et al*.[^58^](#_ENREF_49) | 2005 | 51, M | HCQ, 400 | 1-2. | GHD | necrotizing, vacuolar myopathy rimmed vacuoles |  | Symptoms recovery after 1 m of HQC discontinuation |
| Siddiqui *et al*.[^59^](#_ENREF_50) | 2007 | 88, F | HCQ, 300 | 5 | RA | necrotizing vacuolar myopathy  with curvilinear bodies |  | HQC was discontinued patient died due ventilatory failure |
| Abdel-Hamid *et al*.*[^15^](#_ENREF_15) | 2008 | 56, F | HCQ, 200 | 0.5 | SLE, SS | rimmed vacuoles lysosomal degradation  fiber degeneration |  | HQC discontinuation 2 weeks after patient died |
| Abdel-Hamid *et al*.*[^15^](#_ENREF_15) | 2008 | 64, F | HCQ,200 | 4 | SS | rimmed vacuoles lysosomal degradation fiber degeneration | Elevated | HQC discontinuation after hospital admission Patient died |
| Stevens *et al.*^60^ | 2009 | 42, M | HCQ, 1000 | 15-20 | DL | lysosomal degradation rimmed vacuoles inflammation type I and II fiber atrophy | Elevated | Symptoms recovery after 1m of HQC discontinuation |
| Kwon *et al*.[^61^](#_ENREF_52) | 2010 | 70, F | HCQ, 400 | 5 | RA | Vacuolar myopathy and autophagic vacuoles | Elevated | Symptoms recovery within 18 m of HQC discontinuation |
| Ghosh *et al*.[^62^](#_ENREF_53) | 2013 | 58, F | HCQ, 400 | 15 | NR | Vacuolar myopathy and autophagic vacuoles | Elevated | Symptoms recovery after HQC discontinuation |
| Albay *et al*.[^63^](#_ENREF_54) | 2005 | 56, F | CQ,  *low doses for 13 m 500mg for 5 m | 1.5 | RA | diffuse type II fiber atrophy vacuolar myopathy curvilinear bodies | NR | Symptoms recovery within 1 y of QC discontinuation |
| Posada^64^ | 2011 | 59, F | CQ, 250mg for 7 m 500mg for 7 m | 1.2 | DL, LP | autophagic vacuoles and fiber degeneration | Elevated | Recovery of symptoms and CK normal levels after QC discontinuation |
| Azimian *et al*.*[^65^](#_ENREF_56) | 2012 | 64, F | HCQ, 400 | 20 | RA | necrotizing vacuolar myopathy (quadriceps) | Elevated | Patient died with respiratory failure a day later of HQC discontinuation |
| Azimian *et al*.*[^65^](#_ENREF_56) | 2012 | 38, F | CQ, 500 | 7 | RA | severe vacuolar myopathy curvilinear bodies | Elevated | Patient died 5 days  later after QC  discontinuation |
| Vinciguerra *et al*.[^66^](#_ENREF_57) | 2015 | 63, F | HCQ, 400 | 2 m | RA | diffuse subsarcolemmal accumulation of lipofuscin-like material | Normal | Symptoms recovery within 2 weeks of HQC discontinuation |
| Jafri *et al*.[^67^](#_ENREF_58) | 2017 | 26, F | HCQ, 400 | 1 y | SLE | HQC-induced myopathy with concomitant inflammatory myopathy | NR | Symptoms recovery with HQC discontinuation |
| Shukla *et al*.[^68^](#_ENREF_59) | 2019 | 65, F | HCQ, 200 | 3 y | CTD | vacuolar myopathy withrimmed vacuole (vastus lateralis) | Elevated | Symptoms partially recovered within 9 months after HQC discontinuation - CK normal levels |

F = female, M= male, SLE = Systemic Lupus Eritematosus, RA = rheumatoid arthritis, PM = polymyositis, Sjogren syndrome = SjS; Systemic Sclerosis=SS; discoid lupus=DL; lupus panniculitis = LP; Connective tissue disorder = CTD, Chronic polyarthritis = CPA, graft-versus-host disease = GHD, y= year, m=month. Not reported = NR Symbols * in the author's name indicate studies with more than one patient
